# Transarterial Microembolization in Refractory Plantar Fasciitis: Functional and Patient-Reported Early- to Midterm Outcomes from a Single-Center Pilot Study

**DOI:** 10.3390/diagnostics16142217

**Published:** 2026-07-16

**Authors:** Hüseyin Saygin Tuna, Benjamin Reichardt, Fahrettin Kucukay, Patrick Haage

**Affiliations:** 1 Department of Radiology, HELIOS University Hospital Wuppertal, University of Witten/Herdecke, Heusnerstr. 40, 42283 Wuppertal, Germany; patrick.haage@helios-gesundheit.de; 2 Department of Interventional Radiology and Neuroradiology, Hochsauerland Hospital, Nordring 37-41, 59821 Arnsberg, Germany; benjamin.reichardt@googlemail.com; 3 Department of Interventional Radiology, Faculty of Medicine, Eskisehir Osmangazi University, Buyukdere Meselik Yerleskesi, 26040 Eskisehir, Turkey; fahrettinkucukay@gmail.com

**Keywords:** plantar fasciitis, transarterial microembolization, imipenem-cilastatin, chronic pain, neovascularization, Foot Function Index, patient-reported outcomes, interventional radiology

## Abstract

**Background/Objectives:** Chronic plantar fasciitis refractory to conservative treatment remains a therapeutic challenge, and minimally invasive options targeting pathologic neovascularization are of growing interest. We evaluated the safety and early- to midterm clinical outcomes of transarterial microembolization in refractory plantar fasciitis, with emphasis on functional and patient-reported outcomes. **Methods:** In this single-center observational pilot study with ambispective data collection, 16 procedures in 13 patients were treated using imipenem/cilastatin. The primary outcome was pain intensity (Numeric Rating Scale, NRS); secondary outcomes included the Foot Function Index (FFI), the Patient Global Impression of Change (PGIC), analgesic use, return to activity, need for further treatment, and complications. **Results:** Technical success was 100%. Median NRS decreased from 10.0 at baseline to 3.0 at 1 month and 0.0 at 3 months, with significant improvement maintained through 18 months. Median FFI improved from 9.42 to near zero, and analgesic use declined. At 12 months, 90.9% of procedures reported clinical improvement. Reintervention was required once (6.3%); only minor complications occurred (12.5%), with no major events. Full return to normal activity was achieved in 68.8%. **Conclusions:** Transarterial microembolization was associated with rapid functional and patient-reported improvement and a favorable safety profile. These exploratory, hypothesis-generating findings warrant confirmation in controlled prospective studies.

## 1. Introduction

Plantar fasciitis is a prevalent musculoskeletal condition characterized by debilitating heel pain that is often recalcitrant to conventional therapeutic modalities [1]. Although plantar fasciitis is self-limited in most patients within 6 to 12 months, a clinically relevant subset develops persistent symptoms despite a variety of conservative treatments—including analgesics, local injections, and shockwave therapy—necessitating exploration of alternative interventional strategies [2]. In the present study, only patients with longstanding symptoms beyond expected spontaneous resolution and with documented failure of structured conservative treatment were considered truly refractory and eligible for transarterial microembolization.

Transarterial microembolization has emerged as a potentially effective, minimally invasive technique for chronic inflammatory conditions by targeting pathologic neovascularization [2,3]. It has been applied to mitigate chronic pain in knee osteoarthritis and persistent pain after total knee arthroplasty by targeting genicular arteries that supply neovascularized tissues [4,5]. The underlying principle involves disrupting the inflammatory cascade and inhibiting the proliferation of unmyelinated nerve fibers that contribute to chronic pain by precisely targeting hypervascularized tissues [6,7]. Application to plantar fasciitis is pertinent given the established role of neovascularization in chronic plantar heel pain. Initial evidence arose from broader tendinopathy and enthesopathy studies, in which transarterial microembolization with imipenem/cilastatin produced pain relief, including in isolated plantar fasciitis cases [8]. Subsequent plantar fasciitis-specific studies reported rapid improvement in pain and function after superselective embolization of medial calcaneal and plantar branches using imipenem/cilastatin [9,10,11], and a larger case series with up to 4 years of follow-up reported durable benefit [12].

Despite this growing interest, the available evidence is derived almost entirely from small retrospective series with heterogeneous methodology and a predominant focus on pain reduction. Critically, the most recent superselective plantar fasciitis embolization series [9,10] did not systematically report standardized functional and patient-centered domains such as functional status, global impression of change, analgesic-use trajectory, and return to normal activity. The main contribution of the present work is therefore not the introduction of transarterial microembolization for plantar fasciitis as a novel concept, but an incremental yet clinically relevant extension of the evidence base: we add prespecified, standardized functional and patient-reported outcome measures (Foot Function Index, Patient Global Impression of Change, analgesic-use trajectory, and return to normal activity) together with detailed procedural and radiation-dose data and procedure-level midterm follow-up in a heavily pretreated refractory cohort. These clinically oriented parameters are particularly relevant for interventional radiologists involved in treatment decision-making for chronic musculoskeletal pain.

## 2. Materials and Methods

### 2.1. Study Design

This single-center observational pilot study evaluated the safety and clinical outcomes of transarterial microembolization for therapy-refractory plantar fasciitis in routine clinical practice. Because eligible patients were treated consecutively during the study period and outcome data were subsequently collected at predefined intervals using a standardized questionnaire, data collection was ambispective: treatment was delivered as part of routine care, and structured outcome assessment accrued over the follow-up period, with the analysis performed retrospectively on the assembled dataset. All consecutive patients treated during the study period were screened, and all those meeting eligibility criteria were included to minimize selection bias; no eligible patient was excluded for reasons other than the predefined exclusion criteria. The study period ranged from August 2023 to December 2025. All outcome measures and analytical definitions were specified before data analysis. Reporting adheres to the STROBE guideline for observational cohort studies. Ethics approval and consent details are provided in the Institutional Review Board Statement and Informed Consent Statement.

### 2.2. Patients

Between August 2023 and December 2025, 16 procedures in 13 consecutive patients with chronic plantar fasciitis underwent transarterial microembolization and were included. The unit of analysis was the procedure. Data were extracted from medical records and standardized follow-up forms. Three patients underwent bilateral procedures, accounting for 6 of the 16 procedures; in these patients, the two feet were treated in separate staged sessions (interval approximately 1–2 months) rather than simultaneously, with contralateral treatment performed after clinical reassessment and confirmation of persistent contralateral symptoms. One patient underwent reintervention because of insufficient initial response, performed approximately 3 months after the index procedure. Because of the bilateral cases and the single reintervention, four procedure pairs share a common patient, and complete statistical independence between all observations cannot be fully assumed; this is addressed in the Statistical Analysis and acknowledged as a limitation.

### 2.3. Inclusion and Exclusion Criteria

Patients were eligible if they met all of the following criteria:

• Chronic plantar heel pain, clinically consistent with plantar fasciitis;
• Failure of structured conservative treatment, defined as persistent symptoms despite ≥3 months of physical therapy and/or orthotic use plus at least one additional modality (shockwave, corticosteroid, or platelet-rich plasma injection);
• Clinical diagnosis confirmed by imaging (radiography, ultrasonography, and/or MRI showing fascial thickening >4 mm and/or perifascial edema);
• Availability of baseline assessment and at least one post-interventional follow-up assessment.

Patients were excluded in the presence of any of the following: an alternative primary cause of heel pain; acute infection in the treatment region; clinically relevant peripheral arterial insufficiency; prior surgery in the affected region; or incomplete baseline clinical data. During the study period, one patient assessed for eligibility was excluded because of clinically relevant peripheral arterial insufficiency; accordingly, 14 patients were assessed, and 13 were included. The duration and intensity of conservative therapy prior to referral were determined by the referring physician and were not standardized. Of the 16 procedures, 8 were performed in patients with MRI-based confirmation, 5 with radiography-based confirmation, and 3 with both modalities. Imaging findings were not systematically correlated with clinical outcomes.

### 2.4. Procedure

All procedures were performed in an angiography suite by a single experienced interventional radiologist under local anesthesia. Vascular access was obtained via an antegrade common femoral artery approach using a 4-French sheath. Initial catheterization used a 4-French vertebral catheter (Tempo, Cordis, Mumbai, Maharashtra), followed by superselective catheterization with a 1.7- or 1.9-French microcatheter (Progreat Lambda, Terumo, Tokyo, Japan); a 0.014-inch microwire (Synchro Standard, Stryker, Tijuana, Mexico) was used when required for distal navigation. Selective angiography of the tibial and pedal arteries was performed, and pathologic hypervascularity at the plantar fascia insertion was identified angiographically. Supplying branches—primarily from the medial calcaneal and plantar region—were selectively catheterized.

Imipenem/cilastatin (500 mg/500 mg; Fresenius Kabi, Pune, Maharashtra) was prepared as a suspension with 5 mL iodinated contrast medium and 5 mL normal saline immediately before injection. Upon mixing, imipenem/cilastatin forms a fine particulate suspension with reported particle sizes of approximately 10–100 µm. The proposed mechanism involves transient occlusion of neovessels at the capillary and arteriolar level, disrupting the vascular supply to hypervascularized tissue; as a biodegradable agent, it undergoes gradual degradation, which may permit partial recanalization over time and may contribute to the durability profile observed in this and prior studies [8,12]. Embolization was performed until near stasis or marked reduction in the abnormal vascular blush at the level of the pathologic neovessels. The injected suspension volume ranged from 1 to 4 mL (mean, 2.3 mL), with higher volumes used when multiple supplying branches (typically 2–3 per foot) showed pathologic hypervascularity. Injection was titrated to the angiographic endpoint of flow reduction rather than to a fixed volume per branch.

Procedural radiation parameters were extracted retrospectively from examination protocols. Median fluoroscopy time was 17.2 min (IQR, 9.6–31.9), median dose-area product was 2.96 Gy·cm^2^ (IQR, 1.98–7.71), and median maximum skin entrance dose was 31.2 mGy (IQR, 14.7–78.6). Total procedure time was not systematically documented. Total iodinated contrast medium volume ranged from approximately 20 to 60 mL per procedure, including diagnostic angiography and the contrast component of the imipenem/cilastatin suspension. Higher radiation parameters were observed in three procedures in which obtaining ipsilateral antegrade femoral access required prolonged fluoroscopy of the groin region due to unfavorable body habitus; a contralateral cross-over approach was deliberately avoided to minimize catheter navigation distance to the distal pedal circulation.

The primary target vessel was the posterior tibial artery and its branches; in selected cases, contributing branches from the peroneal artery were also embolized. To minimize non-target embolization, continuous flushing with heparinized saline (1000 IU heparin and 1.4 mg nimodipine in 1000 mL saline), stable distal catheter positioning, and slow, controlled injection under fluoroscopic guidance were applied. Nimodipine was routinely added to the flush as an institutional standard to reduce catheter-induced vasospasm in the distal pedal circulation, and embolization was stopped at near stasis to avoid reflux. Technical endpoints were defined as selective embolization of all identified hypervascular branches with reduction or disappearance of the abnormal blush, and technical success was defined as achievement of these endpoints. After the procedure, local cooling was applied for approximately 18 h and analgesics (paracetamol as needed) were prescribed. Patients were advised to avoid excessive weight-bearing for the first 48–72 h and to gradually resume normal ambulation as tolerated. No structured physiotherapy or formal post-procedural rehabilitation protocol was implemented; patients continued their pre-procedural orthotic use and footwear adaptations at their discretion. The absence of a standardized rehabilitation pathway may have influenced functional recovery and is acknowledged as a limitation.

### 2.5. Outcome Measures

The primary outcome was pain intensity assessed using the Numeric Rating Scale (NRS). Secondary outcomes included functional status (Foot Function Index, FFI, calculated as a mean-based 0–10 score); patient-reported global improvement (Patient Global Impression of Change, PGIC); analgesic use over time, categorized as 0 (none), 1 (occasional NSAID), 2 (daily NSAID), and 3 (opioid); need for further conservative treatment; reintervention; treatment recommendation; return to normal activity (categorized as fully possible, partially improved, or unchanged); and complications. All outcome measures were collected using a standardized questionnaire.

NRS was recorded on a 0–10 scale, with higher values indicating more severe pain, reflecting the maximum pain intensity during weight-bearing activities (first-step pain, prolonged standing, and walking) over the previous week; the maximum-pain rating was chosen rather than average pain to capture the most disabling aspect of plantar fasciitis in this heavily refractory population. FFI was recorded as a mean-item 0–10 score, with higher values indicating worse functional impairment; this mean-item presentation was used to facilitate direct comparison with the NRS, although the use of a modified rather than the original 0–100% visual-analog summation format is acknowledged as a limitation regarding comparability with prior FFI literature. PGIC was assessed using a four-category scale: A (“much better”), near-complete resolution with a very large clinically important change; B (“significantly better”), substantial but incomplete resolution; C (“somewhat better”), a small but noticeable improvement; and D (“unchanged”). The four-category modification simplified patient reporting in a chronic-pain population but reduces sensitivity to small intermediate changes and limits comparability with prior PGIC-based studies. The analgesic-use scale was developed pragmatically for this study and has not been formally validated; it should be interpreted as an exploratory indicator of analgesic burden.

### 2.6. Follow-Up

Follow-up was performed using a standardized questionnaire at 1, 3, 6, 12, 18, and 24 months after transarterial microembolization, and included pain severity, functional outcome, patient-reported improvement, analgesic use, need for additional treatment, return to normal activity, and complications. No cases were lost to follow-up within the first three months. Follow-up availability for NRS was 16 of 16 procedures at 1 and 3 months, 14 of 16 at 6 months, 11 of 16 at 12 months, 8 of 16 at 18 months, and 6 of 16 at 24 months (corresponding to 13, 12, 10, 7, and 6 evaluable patients, respectively). Importantly, the predominant reason for reduced availability at later time points was administrative censoring rather than patient dropout: because enrollment continued throughout the study period, procedures performed later had not yet reached the 18- or 24-month milestone at the time of data extraction. True loss to follow-up (patient disengagement) accounted for only a minority of missing assessments. This distinction is relevant when interpreting later time points, although attrition-related bias cannot be entirely excluded.

### 2.7. Statistical Analysis

Continuous and ordinal variables were summarized as median and interquartile range (IQR), given the small sample size and expected non-normal distribution; categorical variables were reported as number and percentage with exact binomial 95% confidence intervals (CIs) where appropriate. Changes in NRS, FFI, and analgesic-use scores between baseline and each follow-up time point were analyzed using the Wilcoxon signed-rank test for paired samples, with paired available-case analysis at each time point owing to decreasing follow-up availability. Approximate 95% CIs for the median paired change from baseline were derived using the Hodges–Lehmann estimator. Because the three primary score outcomes improved in a near-uniformly concordant direction in nearly all evaluable procedures, the Wilcoxon test statistic—and hence the resulting *p* value—was driven primarily by the number of evaluable pairs at each time point; the *p* values for NRS, FFI, and analgesic use therefore coincide at each time point and should not be interpreted as independent confirmations of effect. Accordingly, all reported *p*-values and confidence intervals are presented as descriptive indicators of the magnitude and consistency of the observed within-cohort changes and should not be interpreted as confirmatory hypothesis tests.

PGIC and return-to-activity outcomes were summarized descriptively at each time point. PGIC categories A and B (“much better” and “significantly better”) were combined to define overall clinical improvement, reported as a proportion with an exact binomial 95% CI. Because PGIC is a change-from-baseline instrument, no inferential test against a baseline state was performed; overall improvement is presented descriptively only. Binary secondary outcomes (need for further treatment, reintervention, treatment recommendation, return to normal activity, and complications) were summarized as numbers and percentages with exact binomial 95% CIs.

Missing data were not imputed, and no correction for multiple testing was applied. Because bilateral procedures and one repeat intervention were included, complete statistical independence between all observations cannot be assumed; therefore, all analyses are interpreted as exploratory and hypothesis-generating rather than confirmatory. All tests were two-sided, and *p* < 0.05 was used as a descriptive threshold rather than a formal inferential criterion. To address non-independence, a prespecified sensitivity analysis restricted to one procedure per patient (*n* = 13) was performed (first treated foot for bilateral cases; index procedure for the reintervention case), with NRS, FFI, and analgesic-use changes re-analyzed using the Wilcoxon signed-rank test and compared with the procedure-level analysis. To evaluate clinical relevance, the proportion of procedures achieving the minimum clinically important difference (MCID) was calculated at each time point, using a ≥2-point NRS reduction and a ≥30% FFI reduction from baseline, consistent with published thresholds for chronic musculoskeletal pain and for foot and ankle outcome measures.

## 3. Results

**Figure 1:**
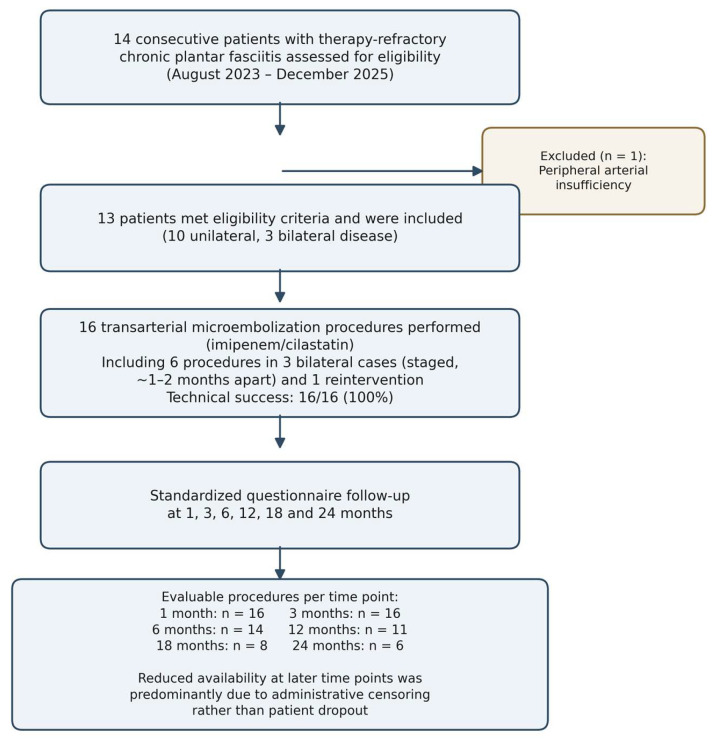
Study flow diagram summarizing patient screening, eligibility assessment, inclusion, transarterial microembolization procedures, and the number of evaluable procedures at each follow-up time point. Reduced availability at later time points was predominantly attributable to administrative censoring rather than to patient dropout.

A total of 16 procedures in 13 patients underwent transarterial microembolization for refractory plantar fasciitis. Technical success was achieved in all procedures (16/16, 100%). The cohort was heavily pretreated and had longstanding symptoms: median age was 51.5 years (IQR, 45.3–58.8), 10 of 13 patients (76.9%) were female, median symptom duration was 15 months (IQR, 12–48), and 9 of 16 procedures (56.3%) involved symptoms lasting longer than 12 months. Prior conservative treatments and baseline scores are summarized in Table 1. The flow of patient screening, eligibility assessment, inclusion, treatment, and follow-up availability is summarized in Figure 1.

**Figure 2:**
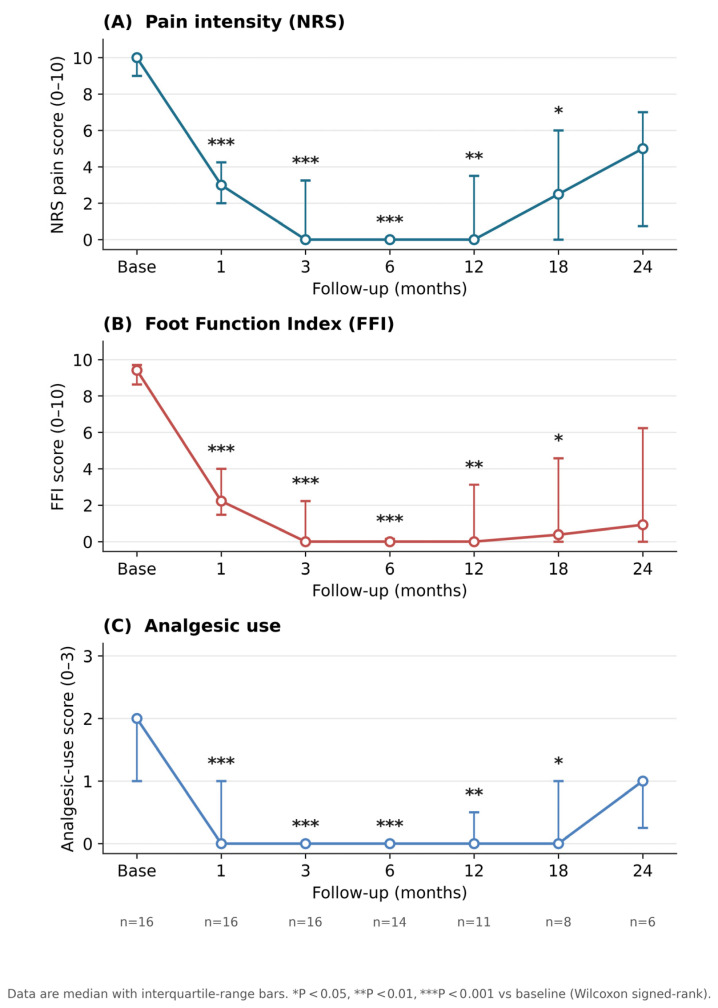
Longitudinal changes after transarterial microembolization for refractory plantar fasciitis. (**A**) Median Numeric Rating Scale (NRS) pain score; (**B**) median Foot Function Index (FFI) score; (**C**) median analgesic-use score. Data are medians with interquartile-range bars. Asterisks indicate statistically significant differences versus baseline (* *p* < 0.05, ** *p* < 0.01, *** *p* < 0.001; Wilcoxon signed-rank test). The number of evaluable procedures at each time point is shown in panel (**C**).

Pain intensity improved substantially after transarterial microembolization (Figure 2A). Median baseline NRS was 10.0 (IQR, 9.0–10.0), decreasing to 3.0 (IQR, 2.0–4.3) at 1 month and 0.0 (IQR, 0.0–3.3) at 3 months, and remaining 0.0 at 6 and 12 months, with a mild increase to 2.5 at 18 months and 5.0 at 24 months. Improvement remained evident through 18 months in the available follow-up cohort (*p* < 0.001 at 1, 3, and 6 months; *p* = 0.002 at 12 months; *p* = 0.016 at 18 months) but not at 24 months (*p* = 0.063); interpretation at 24 months is limited by the small number of evaluable procedures (*n* = 6). The MCID for NRS (≥2-point reduction) was achieved in 15/16 (93.8%) at 1 month, 14/16 (87.5%) at 3 months, 12/14 (85.7%) at 6 months, 10/11 (90.9%) at 12 months, 7/8 (87.5%) at 18 months, and 5/6 (83.3%) at 24 months.

Functional status assessed by FFI also improved (Figure 2B). Median FFI decreased from 9.42 (IQR, 8.63–9.71) at baseline to 2.23 (IQR, 1.48–4.00) at 1 month and 0.0 (IQR, 0.0–2.23) at 3 months, remaining close to zero through 12 months and increasing slightly thereafter (significance pattern identical to NRS). The MCID for FFI (≥30% reduction) was reached in 14/16 (87.5%) at 1 month, 14/16 (87.5%) at 3 months, 11/13 (84.6%) at 6 months, 10/11 (90.9%) at 12 months, 7/7 (100%) at 18 months, and 5/5 (100%) at 24 months. Analgesic use declined over time (Figure 2C): the median analgesic-use score fell from 2.0 (IQR, 1.0–2.0) at baseline to 0.0 through 18 months, with a slight increase to 1.0 (IQR, 0.25–1.0) at 24 months. Detailed values, paired-change CIs, and *p* values are provided in Table 2.

In the prespecified sensitivity analysis restricted to one procedure per patient (*n* = 13), the direction and magnitude of effects were consistent with the procedure-level analysis: median NRS decreased from 10.0 at baseline to 4.0 at 1 month, 1.0 at 3 months, and 0.0 at 6 months (*p* ≤ 0.004 through 12 months; *p* = 0.063 at 18 months; *p* = 0.250 at 24 months), and FFI and analgesic-use scores showed comparable patterns. The reduced statistical significance at later time points in the sensitivity analysis reflects the smaller number of evaluable patients rather than a reversal of effect. Detailed procedure-level values are presented in Table 2.

**Figure 3:**
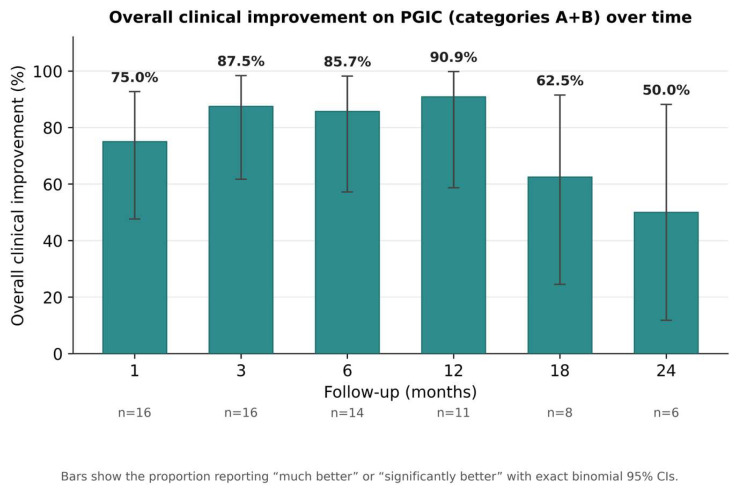
Overall clinical improvement on the Patient Global Impression of Change (PGIC) over time. Bars show the proportion of procedures reporting “much better” or “significantly better” (categories A and B combined) with exact binomial 95% confidence intervals. The number of evaluable procedures at each time point is shown below the x-axis.

Patient-reported global improvement was favorable (Figure 3). The proportion of procedures reporting being “much better” or “significantly better” on the PGIC increased from 75.0% (95% CI, 47.6–92.7) at 1 month to a peak of 90.9% (95% CI, 58.7–99.8) at 12 months, then declined to 62.5% at 18 months and 50.0% at 24 months, paralleling the late attrition and partial loss of effect. Full PGIC category distributions with exact binomial CIs are provided in Table 3.

Regarding additional clinical outcomes, only 3 of 16 procedures (18.8%) required further conservative treatment after transarterial microembolization, whereas 13 of 16 (81.3%) required no further treatment, and reintervention was necessary in 1 procedure (6.3%). Among the 16 evaluable procedures, 12 (75.0%) would recommend the treatment, 1 (6.3%) would not, and 3 (18.8%) were unsure. Return to normal activity—defined as unrestricted resumption of all pre-symptomatic daily activities (including occupational tasks, recreational walking, prolonged standing, and recreational sport at the individual pre-symptom level)—was fully possible in 11 procedures (68.8%), partially improved in 3 (18.8%), and unchanged in 2 (12.5%) (Table 4).

**Figure 4:**
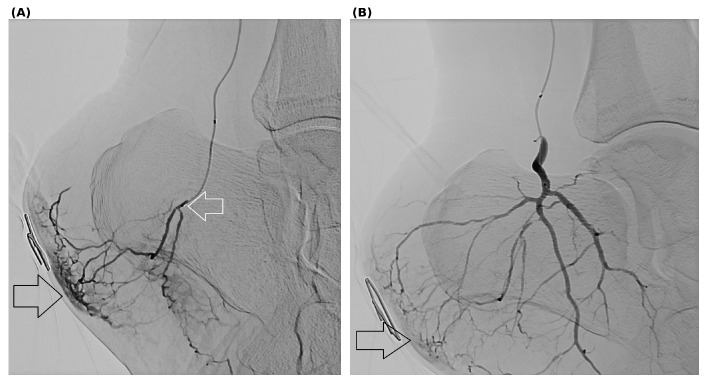
Representative angiographic findings in a procedure for therapy-refractory plantar fasciitis. (**A**) Selective angiography of the medial calcaneal branch (white arrow) demonstrates focal hypervascularity and abnormal neovessel formation at the plantar fascial insertion on the calcaneus (black arrow), corresponding to the symptomatic region. (**B**) After transarterial microembolization, a marked reduction in the abnormal hypervascular blush and decreased visualization of the pathologic neovessels (black arrow) are seen, while flow in the main arterial branches supplying the foot is preserved.

The procedure demonstrated a favorable safety profile (Figure 4). Minor complications occurred in 2 of 16 procedures (12.5%), both small self-limited groin hematomas at the femoral access site that resolved without intervention. No transient skin discoloration of the treated foot, plantar pain flare requiring additional analgesia, ulceration, paresthesia, clinically apparent tissue ischemia, or non-target embolization was observed. No major complications occurred.

## 4. Discussion

This study suggests that transarterial microembolization is associated with rapid and clinically meaningful improvements in pain, functional status, and patient-reported outcomes in patients with refractory plantar fasciitis. Clinical improvement was evident within the first month and remained significant through 18 months. Most procedures resulted in meaningful patient-reported benefit, no need for further treatment, and return to normal activity, suggesting a potential role as a minimally invasive option in selected patients with longstanding plantar fasciitis refractory to conventional therapy. These early observations should nonetheless be regarded as preliminary and hypothesis-generating, given the uncontrolled, single-center design and the small sample size.

The observed reduction in median NRS from 10.0 at baseline to 0.0 at 3–12 months is consistent with prior reports [9,10,11]. Gandhi and Banker reported rapid early pain reduction after embolization using imipenem/cilastatin, with most patients improving within the first 3 months [11]; Tonkaz and Bekci showed that superselective embolization of the medial calcaneal artery can achieve rapid pain relief, supporting the concept that targeting pathologic vascular supply is sufficient to improve symptoms [10]; and Kumar et al. reported favorable short-term outcomes after embolization of branches supplying the painful plantar fascia insertion [9]. The present study extends this literature by incorporating standardized functional recovery and patient-reported outcomes; improvements in return to normal activity, analgesic use, and global patient-reported outcomes provide a more comprehensive assessment of clinical benefit. The pattern of rapid early improvement followed by partial loss of effect in a minority of patients is consistent with the limited available long-term data. Sasaki et al. reported sustained benefit after ultrasound-guided embolization in 66 patients with up to 4 years of follow-up, although some recurrence occurred [12].

Of note, Sasaki et al. employed ultrasound guidance for vessel identification rather than fluoroscopic angiography, which may benefit centers without dedicated angiography suites but may limit assessment of hemodynamic endpoints during embolization [12]. In contrast, the fluoroscopy-guided approach used here allows real-time assessment of flow reduction and precise superselective catheterization of small-caliber branches, at the cost of radiation exposure and the need for specialized equipment. In our cohort, pain relief remained significant through 18 months but not at 24 months, indicating durable but not necessarily permanent symptom control; recurrent neovascularization, mechanical overload, obesity, altered gait, or insufficient rehabilitation may contribute to recurrence. The single procedure in which the patient would not recommend treatment corresponded to the case requiring reintervention, underscoring the importance of counseling patients about the potential for recurrence and repeated intervention in a minority of cases.

The favorable functional outcomes further support clinical relevance. Median FFI improved from 9.42 to near-zero values, and most procedures achieved full return to normal activity, with only a small proportion requiring further treatment or reintervention. Because previous studies focused largely on pain scores, the inclusion of FFI, PGIC, return-to-activity measures, and analgesic use represents a strength of this work. It should nonetheless be emphasized that the FFI and PGIC were applied in adapted forms (a mean-item 0–10 FFI rather than the original 0–100% summation, and a four-category PGIC), which may limit direct numerical comparison with studies that used the original instruments. Given the decreasing follow-up availability at later time points, particularly at 24 months, long-term outcomes should be interpreted cautiously; although available-case analysis demonstrated persistent improvement in a subset of procedures, attrition-related overestimation of treatment effect cannot be entirely excluded. The reduction in analgesic requirements is also clinically meaningful: all procedures involved prior NSAID use and some required opioids, whereas analgesic use decreased to near zero in most cases and remained low through 18 months, which may also reduce risks associated with long-term analgesic therapy.

The underlying mechanism likely involves occlusion of abnormal neovessels and interruption of associated inflammatory and neural pathways. Histopathologic and imaging studies have shown that chronic tendinopathies—and, similarly, plantar fasciitis—are associated with neovascularization and proliferation of unmyelinated sensory nerve fibers [6,7,8]; by targeting these hypervascular regions, transarterial microembolization may reduce inflammation and nociceptive signaling, and the use of imipenem/cilastatin as a temporary embolic agent may permit transient occlusion while minimizing prolonged ischemia. The marked pain relief observed in several procedures, including reduction from NRS 10 to 0, warrants consideration of contributing factors beyond the procedural effect. The very high and homogeneous baseline NRS values reflect a heavily refractory cohort but also raise the possibility of regression to the mean, as patients reporting extreme baseline pain are statistically more likely to report lower values subsequently. Selection bias is possible, since patients with the most severe symptoms may be preferentially referred for interventional treatment, and natural disease fluctuation, concomitant lifestyle modification, reduced weight-bearing, or a placebo response cannot be excluded in the absence of a control group. Nevertheless, the rapid onset of improvement within the first month, combined with parallel reductions in analgesic use and functional gains, is most consistent with a procedural mechanism rather than spontaneous remission, which in refractory plantar fasciitis typically follows a more gradual course.

Beyond imipenem/cilastatin, alternative embolic agents have been explored in musculoskeletal embolization, including calibrated microspheres of varying sizes (typically 75–150 µm or 100–300 µm). These particles offer predictable occlusion depth but carry a higher risk of permanent vessel occlusion and non-target embolization than imipenem/cilastatin, a temporary embolic agent; the choice of embolic material may influence both durability and safety, although comparative data in plantar fasciitis specifically remain absent. The safety findings are encouraging and consistent with the broader musculoskeletal embolization literature: technical success was achieved in all procedures, minor complications occurred in two, and no major complications were observed, with similar profiles reported in plantar fasciitis and in conditions such as knee osteoarthritis and Achilles tendinopathy [3,4,5]. Given the delicate distal arterial network of the plantar foot, careful superselective catheterization remains essential to minimize risks such as non-target embolization or tissue ischemia.

Other investigators have documented imaging-detectable anatomical changes in the plantar fascia after treatment. On magnetic resonance imaging, significant reductions in plantar fascia thickness, high-signal-intensity change, and perifascial and bone-marrow edema have been reported following conservative modalities such as extracorporeal shockwave therapy, low-level laser therapy, and therapeutic ultrasound [13]. Longitudinal ultrasonographic and sonoelastographic follow-up extending to 12 months has similarly shown persistent reductions in fascial thickness together with morphological and elasticity changes that parallel clinical improvement [14]. Comparable post-treatment imaging changes have been described after transcatheter arterial embolization in other musculoskeletal indications, in which MR imaging demonstrated regression of pathologic tissue changes after embolization [3]. In the present cohort, standardized pre- and post-procedural imaging was not obtained, and we therefore could not confirm whether transarterial microembolization produced analogous structural remodeling of the plantar fascia. Incorporating systematic ultrasonography or MRI at longer-term milestones, including the 18- and 24-month time points, in future studies would allow the durability of clinical benefit to be correlated with objective anatomical changes in the plantar fascia.

This study has several limitations. First, the single-center design with ambispective data collection and a small cohort may introduce selection bias and limit generalizability. Second, the absence of a control group precludes differentiation between treatment effects and natural disease course or placebo response. Third, follow-up availability declined over time, particularly at 24 months; although this was predominantly due to administrative censoring rather than dropout, the possibility that patients with better outcomes disengaged, or that patients with worse outcomes sought care elsewhere, cannot be excluded. Fourth, imaging findings were not systematically correlated with clinical outcomes, and no post-procedural imaging was obtained to objectively verify the intended biological effect on pathologic neovascularity. Fifth, because some procedures are derived from bilateral disease or repeat intervention in the same patient, complete statistical independence cannot be assumed; the prespecified sensitivity analysis restricted to one procedure per patient (*n* = 13) showed consistent results, but the single reintervention is itself counted among the 16 procedures and contributes its own outcome data, introducing a degree of circularity in procedure-level reintervention estimates. In addition, in bilateral cases, the baseline assessment of the second-treated foot was obtained after the first procedure, and return to normal activity—an inherently patient-level outcome—was recorded per procedure, which may further limit the independence and interpretation of the paired comparisons. Sixth, several outcome instruments were modified (a mean-item FFI rather than the original 0–100% format and a four-category PGIC), and the analgesic-use and willingness-to-recommend measures were non-validated, limiting comparability with prior literature; reporting the FFI in its original format in future studies would improve direct comparability. Finally, no standardized post-procedural rehabilitation protocol was implemented, and no correction for multiple testing was applied; however, the consistency of improvement across multiple outcome domains supports a clinically meaningful treatment effect.

Future studies should include larger multicenter prospective cohorts and randomized controlled trials comparing transarterial microembolization with established therapies such as corticosteroid injection, platelet-rich plasma, extracorporeal shockwave therapy, or radiofrequency ablation. Standardized patient selection, validated outcome instruments, and systematic pre- and post-procedural imaging (ultrasonography, MRI, or magnetic resonance angiography) may help identify predictors of response, optimize patient selection and technique, and assess long-term durability.

## 5. Conclusions

In this single-center pilot study, transarterial microembolization using imipenem/cilastatin was associated with rapid improvements in pain, function, and patient-reported outcomes, reduced analgesic use, and a favorable safety profile in patients with refractory plantar fasciitis. These findings suggest a potential role as a minimally invasive treatment option in selected patients but should be regarded as exploratory and hypothesis-generating. Larger prospective, controlled, multicenter studies with standardized protocols, validated outcome measures, and extended follow-up are needed to confirm these preliminary observations.

## Figures and Tables

**Table 1:** Baseline demographic and clinical characteristics of the study population. Continuous variables are presented as median (IQR) and categorical variables as numbers (%). Demographic variables (age, sex, side of disease) are reported at the patient level (*n* = 13); prior treatments, baseline scores, and technical success are reported at the procedure level (*n* = 16).

Variable	Value
Number of patients/procedures	13/16
Age, years	51.5 (45.3–58.8)
Sex (patients), male/female	3/10
Symptom duration, months	15 (12–48)
Side of disease (patients), unilateral/bilateral	10/3
Number of reinterventions	1
Baseline NRS score	10.0 (9.0–10.0)
Baseline FFI score	9.42 (8.63–9.71)
Baseline analgesic-use score	2.0 (1.0–2.0)
Prior physical therapy	13 (81.3%)
Prior orthotic/insole use	11 (68.8%)
Prior corticosteroid injection	8 (50.0%)
Prior platelet-rich plasma injection	3 (18.8%)
Prior extracorporeal shockwave therapy	5 (31.3%)
Prior NSAID use	16 (100%)
Prior opioid use	3 (18.8%)
Symptom duration >12 months	9 (56.3%)
Technical success	16/16 (100%)

**Table 2:** Pain intensity, functional outcome, and analgesic use over time after transarterial microembolization. Values are median (IQR). The 95% CI column reflects the Hodges–Lehmann estimate of the median paired change from baseline. *p* values were calculated using the Wilcoxon signed-rank test versus baseline; identical *p* values across the three outcomes reflect the near-uniform direction of change and the number of evaluable pairs (see Section 2.7). The number of evaluable procedures (*n*) is indicated in the first column.

Time Point	NRS Median (IQR)	95% CI (Change)	*p*	FFI Median (IQR)	95% CI (Change)	*p*	Analgesic Median (IQR)	95% CI (Change)	*p*
Baseline (*n* = 16)	10.0 (9.0–10.0)	—	—	9.42 (8.63–9.71)	—	—	2.0 (1.0–2.0)	—	—
1 month (*n* = 16)	3.0 (2.0–4.25)	−7.0 to −5.0	<0.001	2.23 (1.48–4.00)	−7.20 to −4.90	<0.001	0.0 (0.0–1.0)	−2.0 to −1.0	<0.001
3 months (*n* = 16)	0.0 (0.0–3.25)	−9.0 to −7.0	<0.001	0.0 (0.0–2.23)	−8.80 to −7.10	<0.001	0.0 (0.0–0.0)	−2.0 to −1.0	<0.001
6 months (*n* = 14)	0.0 (0.0–0.0)	−10.0 to −8.0	<0.001	0.0 (0.0–0.15)	−9.10 to −7.80	<0.001	0.0 (0.0–0.0)	−2.0 to −2.0	<0.001
12 months (*n* = 11)	0.0 (0.0–3.5)	−9.0 to −6.0	0.002	0.0 (0.0–3.12)	−8.70 to −6.40	0.002	0.0 (0.0–0.5)	−2.0 to −1.0	0.002
18 months (*n* = 8)	2.5 (0.0–6.0)	−9.0 to −3.0	0.016	0.38 (0.0–4.58)	−7.10 to −2.90	0.016	0.0 (0.0–1.0)	−2.0 to −1.0	0.016
24 months (*n* = 6)	5.0 (0.75–7.0)	−8.0 to 0.0	0.063	0.92 (0.0–6.23)	−6.50 to 0.0	0.063	1.0 (0.25–1.0)	−2.0 to 0.0	0.063

**Table 3:** Patient Global Impression of Change (PGIC) over time after transarterial microembolization. Values are numbers (%) with exact binomial 95% confidence intervals. Overall clinical improvement was defined as PGIC categories A and B (“much better” and “significantly better”) combined. Because PGIC is a change-from-baseline instrument, results are summarized descriptively without inferential testing against a baseline state. *N* indicates the number of evaluable procedures at each follow-up time point.

PGIC Category	1 Month *n*/*N* (%)	95% CI	3 Months *n*/*N* (%)	95% CI	6 Months *n*/*N* (%)	95% CI	12 Months *n*/*N* (%)	95% CI	18 Months *n*/*N* (%)	95% CI	24 Months *n*/*N* (%)	95% CI
Much better (A)	5/16 (31.2%)	11.0–58.7	11/16 (68.7%)	41.3–89.0	11/14 (78.6%)	49.2–95.3	7/11 (63.6%)	30.8–89.1	5/8 (62.5%)	24.5–91.5	2/6 (33.3%)	4.3–77.7
Significantly better (B)	7/16 (43.7%)	19.8–70.1	3/16 (18.8%)	4.0–45.6	1/14 (7.1%)	0.2–33.9	3/11 (27.3%)	6.0–61.0	0/8 (0%)	0–36.9	1/6 (16.7%)	0.4–64.1
Somewhat better (C)	3/16 (18.8%)	4.0–45.6	0/16 (0%)	0–20.6	0/14 (0%)	0–23.2	0/11 (0%)	0–28.5	2/8 (25.0%)	3.2–65.1	0/6 (0%)	0–45.9
Unchanged (D)	1/16 (6.3%)	0.2–30.2	2/16 (12.5%)	1.6–38.3	2/14 (14.3%)	1.8–42.8	1/11 (9.1%)	0.2–41.3	1/8 (12.5%)	0.3–52.7	3/6 (50.0%)	11.8–88.2
Overall improvement (A + B)	12/16 (75.0%)	47.6–92.7	14/16 (87.5%)	61.7–98.4	12/14 (85.7%)	57.2–98.2	10/11 (90.9%)	58.7–99.8	5/8 (62.5%)	24.5–91.5	3/6 (50.0%)	11.8–88.2

**Table 4:** Patient-reported and clinical outcomes after transarterial microembolization. Values are numbers (%). Binary outcomes were assessed across all 16 procedures; return to activity was assessed at the last available follow-up per procedure.

Variable	Value
Need for further conservative treatment	3/16 (18.8%)
No further treatment required	13/16 (81.3%)
Reintervention	1/16 (6.3%)
Would recommend treatment	12/16 (75.0%)
Would not recommend treatment	1/16 (6.3%)
Unsure about recommendation	3/16 (18.8%)
Full return to normal activity	11/16 (68.8%)
Partial return to normal activity	3/16 (18.8%)
Return to normal activity unchanged	2/16 (12.5%)
Minor complications	2/16 (12.5%)
Major complications	0/16 (0%)

## Data Availability

The datasets generated and analyzed during the current study are available from the corresponding author upon reasonable request. The data are not publicly available due to privacy restrictions.
